# Dyadic Social Interaction as an Alternative Reward to Cocaine

**DOI:** 10.3389/fpsyt.2013.00100

**Published:** 2013-09-12

**Authors:** Gerald Zernig, Kai K. Kummer, Janine M. Prast

**Affiliations:** ^1^Experimental Psychiatry Unit, Department of General Psychiatry and Social Psychiatry, Innsbruck Medical University, Innsbruck, Austria; ^2^Department of Psychology, Leopold-Franzens University of Innsbruck, Innsbruck, Austria

**Keywords:** social interaction, cocaine, conditioned place preference, Sprague-Dawley rat, C57BL/6 mouse, substance use disorder

## Abstract

Individuals suffering from substance use disorders often show severely impaired social interaction, preferring drugs of abuse to the contact with others. Their impaired social interaction is doubly harmful for them as (1) therapy itself is based and dependent on social interaction and as (2) social interaction is not available to them as an “alternative”, i.e., non-drug reward, decreasing their motivation to stop drug use. We therefore developed an animal experimental model to investigate the neurobiology of dyadic social interaction- vs. cocaine reward. We took care to avoid: (a) engaging sexual attraction-related aspects of such a social interaction and (b) hierarchical difference as confounding stimuli. The cocaine- or social interaction stimulus was offered – in a mutually exclusive setting – within the confines of a conditioned place preference (CPP) apparatus. In our paradigm, only four 15-min episodes of social interaction proved sufficient to (i) switch the rats’ preference from cocaine-associated contextual stimuli to social interaction CPP and (ii) inhibit the subsequent reacquisition/reexpression of cocaine CPP. This behavioral effect was paralleled by a reversal of brain activation (i.e., EGR1 expression) in the nucleus accumbens, the central and basolateral amygdala, and the ventral tegmental area. Of relevance for the psychotherapy of addictive disorders, the most rewarding sensory component of the composite stimulus “social interaction” was touch. To test our hypothesis that motivation is encoded in neuron ensembles dedicated to specific reward scenarios, we are currently (1) mapping the neural circuits involved in cocaine- vs. social-interaction reward and (2) adapting our paradigm for C57BL/6 mice to make use of the plethora of transgenic models available in this species.

## Introduction

Individuals suffering from substance use disorders often show severely impaired social interaction, preferring drugs of abuse to the contact with others. Their impaired social interaction is doubly harmful for them as (1) therapy itself is based and dependent on social interaction with their psychotherapist (1, 2), physician, case manager, etc., and as (2) social interaction is not available to them as an “alternative”, i.e., non-drug reward, decreasing their motivation to stop drug use. We therefore developed an animal experimental model to investigate the neurobiology of dyadic social interaction (DSI)- vs. cocaine-reward. The results were very encouraging, robust, and have so far been demonstrated by three generations of experimenters comprising a total of seven individual experimenters (3–6): just four episodes of 15-min DSI with a weight- and sex-matched male conspecific were not only able to countercondition place preference from cocaine to this form of social interaction but, following a subsequent cocaine exposure, were able to inhibit the reacquisition of cocaine conditioned place preference (CPP) that regularly occurred if the rats’ cocaine CPP was simply extinguished without social interaction counterconditioning (SIC). After the four DSI episodes, many animals even fully defended their DSI preference against the cocaine challenge (3). The behavioral reversal was paralleled by a DSI-mediated reversal in the cocaine CPP-induced activation (quantified by EGR1 expression) of several brain regions implicated in reward/reinforcement: the nucleus accumbens shell (AcbSh) and core (AcbC), the central (CeA) and basolateral (BLA) amygdala, and the ventral tegmental area (VTA). We are currently investigating which neuron type(s) in the accumbens mediate this effect and are validating our paradigm in C57BL/6 mice to benefit from transgenic methods for the neurobiological investigation of this therapeutically promising effect. Of note, impaired social interaction is also a hallmark of several other psychiatric disorders such as major depression, dysthymia, and autism spectrum disorders. We initially focused on cocaine as a prototypical drug of abuse and opine that our findings may well generalize to ethanol (alcohol) and opioids. In fact, time allowing, we intend to expand our paradigm to ethanol vs. social interaction. To conclude, a number of patients suffering from a variety of psychiatric disorders would profit from the societally beneficial investigation of the neurobiological basis of the shift of one’s preference from a harmful stimulus to social interaction.

## Methods: Overview of Our Behavioral Paradigm

In the operationalization of the beneficial effect of social interaction on drug craving, we took care (a) to avoid engaging sexual attraction-related aspects of such a social interaction by allowing only same-sex interaction between male conspecifics and (b) to avoid hierarchical difference as a confounding stimulus by allowing DSI only between weight-matched males. The cocaine- or social-interaction stimulus was offered – in a mutually exclusive setting – within the confines of a CPP apparatus. CPP (3–12) is a plausible measure of what humans may be able to report as “drug craving” (13), one of the most important determinants of drug lapse and relapse (14). Of note, CPP has also been demonstrated in humans (15).

In our paradigm, the animals receive an intraperitoneal (i.p.) injection of saline and are placed in the conditioning chamber, either alone (saline control, sal) or with another conspecific of the same-sex and weight (DSI) or receive an i.p. injection of 15 mg/kg cocaine (concentration refers to pure base) and are placed in the conditioning chamber alone (coc). Training- and test session length is always 900 s. Our paradigm comprises three different experimental approaches, ranked according to decreasing experimenter time requirement:

(1) **SIC effect on the reacquisition/reexpression of cocaine CPP**
  This is the experiment in our paradigm that in our opinion has the highest face validity and translational promise for the human situation. It is also by far the most time consuming, requiring 24 days for completion [see fig. 1 of (3)]. Animals are first trained to acquire CPP for cocaine (coc) in an alternate-day design with four exposures to coc or saline (sal) each, with cocaine assigned to the initially non-preferred compartment. After the animal has acquired coc CPP, the preference for coc is extinguished by pairing the previously coc-associated compartment with sal too. Extinction is obtained and tested in four cycles, each consisting of sal conditioning – sal conditioning – CPP test (T1–T4). After T4, the animal is exposed to one more coc training session (arguably modeling a “freebie” in the human situation) and tested for reacquisition/reexpression of coc CPP 24 h later, i.e., in a cocaine-free state. In the SIC condition, after CPP for coc is established, the previously coc-paired compartment is paired with sal, and the previously sal-paired compartment is now paired with the usual i.p. sal injection followed by a DSI with a sex- and weight-matched male conspecific through cycles T1–T4, each cycle consisting of sal – DSI – CPP test. The final coc challenge (i.e., coc training) and the test of the reacquisition/rexpression of coc CPP is performed as described for the coc CPP extinction protocol.
  In one special application of this experimental approach (10), we performed the experiment only to the end of the first reconditioning cycle, i.e., T1. At T1, the animal usually has lost its preference for coc, spending equal amounts of time in the previously coc-paired chamber (now sal-paired), and the DSI-paired chamber (previously paired with sal alone). We hypothesized that any compound that enhances this beneficial reversal from coc CPP to DSI CPP would produce an increased time in the DSI-associated chamber. The sigma1 receptor antagonist BD1047 did (10).
(2) **Concurrent CPP for social interaction vs. cocaine: a choice paradigm**
  In a much less time-consuming experiment [total experiment time, 10 days, see fig. 1 of Ref. (3)], CPP for DSI and cocaine is acquired concurrently in an alternate-day stimulus exposure paradigm (9). It turned out that at a coc dose of 15 mg/kg i.p., CPP for coc and DSI is the same, resulting in no overall preference for either stimulus (9). By lesioning different brain regions, we could tip the CPP balance as if on a seesaw: lesioning the AcbC or BLA shifted net CPP toward social interaction, whereas lesioning the AcbSh shifted net CPP toward cocaine (9). As even the anatomically crude lesioning of whole brain regions produced such a dramatic effect on the net CPP preference, we expect this paradigm to yield data of extreme interest when applying double immunohistochemical methods (see Outlook, below). By manipulating the cocaine training dose, this paradigm also allows for a fully quantitative analysis of the preference shift. Our concurrent drug- vs. social-interaction paradigm has been confirmed and further validated with amphetamine by Bardo and colleagues (16).
(3) **CPP for either social interaction or cocaine**
  The purest experimental approach with respect to the neurobiological investigation of the CPP induced by coc vs. DSI is, of course, to train and test the animals separately for the coc- or the DSI stimulus (12). The time requirement is 10 days [see fig. 1 of Ref. (3)], i.e., the same as for the concurrent CPP paradigm described under item 2, above. Bardo and coworkers have further validated our paradigm and have found that the length of exposure to DSI and the age of the animals are of great importance for successfully establishing DSI as a reward (16).
  Thus, each of our three different experimental approaches yields answers to different questions as detailed below.

## Results: Dyadic Social Interaction vs. Cocaine: Changes in Conditioned Place Preference and Regional Brain Activity as Quantified by EGR1 Mapping

In our paradigm, just four 15-min episodes of social interaction with a weight- and sex-matched male rat not only reversed CPP from cocaine to this form of social interaction and inhibited the subsequent reacquisition/reexpression of cocaine CPP (3), but also reversed the cocaine-conditioning-induced activation, i.e., protein expression of the immediate early gene EGR1 (early growth response 1; also known as zif268), in the AcbSh and AcbC, the VTA, and the BLA and CeA (3, 6). The cocaine CPP-associated EGR1 expression reversal by social interaction was paralleled by an increase in pCREB (the phosphorylated form of cAMP response element binding protein) and a decrease in FosB/deltaFosB expression (5), echoing opposing roles of pCREB vs. deltaFosB in drug reward (17, 18). In a rat concurrent CPP paradigm, lesioning the AcbC, or the BLA tipped the balance toward the acquisition/expression of social interaction CPP, whereas AcbSh lesioning shifted the balance toward cocaine CPP (9), suggesting that the core is more important for acquisition/expression of drug reward and the shell for acquisition/expression of social interaction reward. Differential activation during acquisition/expression of CPP was also demonstrated (12) for the AcbC and prelimbic cortex (DSI CPP) and the granular and dorsal agranular insular cortex (cocaine CPP). The sensory component of the composite stimulus “social interaction” that contributed most to its rewarding effects was touch (taction), whereas crowding diminished social interaction reward (4). When we limited touch by separating the rats by “prison-type” steel bars, it still proved rewarding (4). Our findings were confirmed by Neisewander and colleagues using a steel mesh barrier (19). Increasing the weight of the social interaction partner (thus most likely increasing his physical dominance/hierarchical superiority) systematically decreased the rewarding effect of social interaction, more predictably for the smaller rat than for the bigger rat (4).

## Outlook: Identification of the Involved Neuron Ensembles by Double Immunochemistry

In our paradigm (3), DSI had the most pronounced effect on AcbSh and AcbC activation and on the activation of brain regions containing projection neurons to the accumbens. We therefore want to focus on the accumbens. The major output neurons of the nucleus accumbens, just like of the more dorsal parts of the striatum, are dynorphin/D1-dopamine-receptor-expressing medium spiny neurons (D1-MSNs, i.e., GABAergic projection neurons) and enkephalin/D2-expressing MSNs (D2-MSNs). The activity of these D1- and D2-MSNs is not only regulated by dopaminergic afferents from the VTA but also, directly and indirectly, by a number of different GABAergic interneurons (GAIs), i.e., parvalbumin-positive (PV-GAIs), NPY-positive GAIs (NPY-GAIs), calretinin-positive GAIs (CR-GAIs), by cholinergic interneurons (ChIs) (20–24), and by glutamatergic terminals from the medial prefrontal and orbital cortex and the amygdala (25–31). We intend to quantify the contribution of each of the neuron types described above by double immunohistochemistry for EGR1 and either dynorphin for D1-MSNs, enkephalin or the D2 dopamine receptor (D2DR) for D2-MSNs, parvalbumin (PV-GAIs; fast-spiking), neuropeptide Y (NPY-GAIs; prolonged plateau potential low threshold spiking GAIs, PLTS), calretinin (CR-GAIs), or choline acetyltransferase (ChAT; ChIs).

In order to make use of the plethora of transgenic models available in the mouse, we also intend to validate and optimize our behavioral paradigm in this species, i.e., in C57BL/6 mice. Experiments are ongoing and promising.

## Discussion

Our findings are part of an emerging wealth of data on social interaction and animal experimental measures of substance use disorders, generated by a number of independent groups over the last 6 years (16, 19, 32–34). In contrast to Neisewander and colleagues (32) who showed that social interaction, if offered together with the drug stimulus, actually enhanced drug reward, we demonstrated that social interaction, if offered in a mutually exclusive setting, decreased it. While Izenwasser and colleagues (34) or Ribeiro Do Couto and coworkers (33) studied the effect of dyadic and group social interaction offered in the home cage, we focused on DSI offered as a non-drug stimulus within the confines of the CPP apparatus itself. Independently from us, Neisewander and colleagues demonstrated the rewarding effect of limited physical contact through a mesh barrier (19), in accordance with our observation that physical contact through “prison-type” steel bars was sufficient to produce reward, albeit to a lesser degree than full physical contact (4). Bardo and coworkers have taken great care to further validate our paradigm and have found that the length of exposure to DSI and the age of the animals is of great importance for successfully establishing DSI as a reward (16).

Our choice of EGR1 as a brain activation marker was based on the seminal paper by Everitt and colleagues (35) who showed that infusion of EGR1 antisense oligodeoxynucleotides into the BLA prior to the reactivation of a well-learned memory for a conditioned stimulus-cocaine association, abolished the acquired conditioned reinforcing properties of the drug-associated stimulus and thus its impact on the learning of a new cocaine-seeking response and that reconsolidation of CS-fear memories also required EGR1 in the amygdala.

A great deal of time can be spent on discussing the merits and shortcomings of using a biased approach in CPP, i.e., assigning the to-be conditioned stimulus to the initially non-preferred chamber – as opposed to, say, counterbalancing the animals according to their pretest preference to obtain an across-group equality in pretest times [please, see the excellent reviews of Ref. (7, 8)]. From the translational perspective, it does not matter: substance use disorders are defined by one stimulus, i.e., the drug of abuse, channeling, and limiting the individual’s behavior toward this drug of abuse, regardless of where and how broad the interests of this individuals lay before the drug of abuse took control of the individual’s behavior (36, 37).

With respect to other limitations of our paradigm, our findings, obtained in “young adult” rats, may not be generalizable to “old adult” rats, as the findings of Bardo and colleagues (16) suggest. In our paradigm, we have also tried to increase the attractiveness of DSI by single-housing the animals, another issue addressed in Ref. (16). It also turned out that limiting the time of exposure to social interaction to 15 min may have proved to be favorable to obtain a rewarding effect of this non-drug stimulus (16). We chose to investigate males; applying our paradigm to females is an obvious avenue to explore.

The development of our experimental paradigm was based on the intention of one of us (G.Z.) to operationalize in an animal model what he thinks to be one of the core aspects of the beneficial effects of religious experience, i.e., an idealized dyadic relationship, in recovering addicts (38, 39). While we indeed did find that DSI proved beneficial with respect to the reorientation away from cocaine (as a prototypical drug of abuse) toward social interaction as a non-drug stimulus, there is no agreement that our paradigm indeed operationalizes any aspect of religious experience. Thus, a disputed premise may have led to what we opine is an important experimental finding.

Finally, our findings present a challenge to a view holding that drugs of abuse control an individual to a degree that precludes any choice. Similar to our findings with DSI, Ahmed and coworkers could demonstrate in rats that intense sweetness can surpass cocaine reward, even in drug-sensitized and -addicted individuals (40). The findings of Ahmed and colleagues and our group offer hope for the psychotherapy of drug dependent individuals in that a behavioral change may still be possible, even if an individual initially shows a seemingly exclusive preference for a drug of abuse.

## Conflict of Interest Statement

The authors declare that the research was conducted in the absence of any commercial or financial relationships that could be construed as a potential conflict of interest.
